# 早期非小细胞肺癌淋巴结转移规律及清扫方式研究进展

**DOI:** 10.3779/j.issn.1009-3419.2019.08.07

**Published:** 2019-08-20

**Authors:** 运 陆, 腾 马, 雷 王, 涛 薛

**Affiliations:** 210000 南京，东南大学附属中大医院胸心外科 Department of Cardiothoracic Surgery, the Affiliated Zhongda Hospital of Southeast University, Nanjing 210000, China

**Keywords:** 肺肿瘤, 淋巴结转移, 清扫方式, Lung neoplasms, Lymph node metastasis, Cleaning method

## Abstract

肺癌是目前我国发病率和死亡率均居首位的恶性肿瘤，其中以非小细胞肺癌为主要病理类型。淋巴结转移是非小细胞肺癌最常见和最主要的转移途径，也是影响肺癌分期和预后最重要的因素。由于目前通过现有手段术前很难准确判断早期非小细胞肺癌患者的淋巴结受累情况。因此，在早期非小细胞肺癌中，尤其是在临床Ⅰ期非小细胞肺癌患者中，淋巴结清扫方式一直存在很大争议。本文就非小细胞肺癌淋巴结转移的规律及清扫方式进行综述。

## Abstract (en)

Lung cancer is the malignant tumor with the highest morbidity and mortality in China. Non-small cell lung cancer is the main pathological type. Lymph node metastasis is the most common and primary metastatic pathway in non-small cell lung cancer. Acknowledged as the major factor determining tumor staging and prognosis. Unfortunately, it' s presently hard to make an accurate preoperative judgment on the lymph node metastasis of early stage non-small cell lung cancer. Consequently, there has been a long-standing controversy on lymph node sweeping for early stage non-small cell lung cancer, especially for those clinical stage Ⅰ. The regularity of non-small cell lung cancer lymph node metastasis and the ways of lymph node sweeping will be reviewed in this paper.

肺癌是我国目前发病率和死亡率均居首位的恶性肿瘤，肺癌的主要组织学类型为非小细胞肺癌（non-small cell lung cancer, NSCLC）（80%-85%），其中NSCLC主要分为鳞状细胞癌（25%）、肺腺癌（40%）和大细胞癌^[1]^。淋巴结转移是NSCLC最常见和最主要的转移途径。也是影响分期和预后的最重要因素。因此，准确评估淋巴结的病理状态对于指导手术方法的选择、制定合适的淋巴结清扫方案、判断预后、提高肺癌患者的生存率以及改善生活质量具有重要意义。然而，通过现有手段，术前很难准确判断早期NSCLC患者的淋巴结受累情况。因此，在早期NSCLC中，尤其是在临床Ⅰ期NSCLC患者中，淋巴结清扫方式一直存在很大争议。本文将对国内外NSCLC淋巴结转移规律和淋巴结清扫方式的相关研究结果进行综述。

## 1 NSCLC淋巴结引流路径研究

肺脏淋巴引流主要由两大系统组成，即间质淋巴管和实质淋巴管^[2]^。两个主要淋巴引流系统之间有丰富的吻合支，并且变异较多。肺癌的淋巴结转移也与淋巴引流有关。肺组织产生的淋巴液首先流回段支气管周围的淋巴结，然后沿着段间流回叶门，然后通过叶间流回肺门。来自右上叶的淋巴液主要通过气管旁通路和静脉间隙通路注入右颈静脉角，一部分淋巴液回流至隆突下淋巴结，然后沿着右两条通路上升。左上叶的淋巴液主要沿动脉间隙和气管旁通道向上流动，而另一部分先回流至隆突下的淋巴结，然后沿左侧两条通道上行，最后流入左颈静脉角。来自一些舌段的淋巴液可以流回食管旁淋巴结和下肺韧带的淋巴结。来自右中、下叶和左下叶的淋巴液可以首先回流至食管旁淋巴结和下肺韧带淋巴结，向上注入隆突下淋巴结，或者直接回流至隆突下淋巴结，然后分别沿左右两条路径向上注入右颈静脉角和左颈静脉角。研究^[3]^表明，在左下叶的淋巴液注入隆突下淋巴结后，相当一部分淋巴液沿着右侧两条通道注入右颈静脉角。除了两肺下叶的淋巴液向上回流外，一部分淋巴液通过膈肌注入腹腔淋巴结。

## 2 NSCLC淋巴结转移影响因素

NSCLC淋巴结转移较为常见，其转移规律一般是“自上而下，由远及近，由肺内到肺外”，掌握肺癌淋巴结转移规律，充分了解其特点，有助于术中彻底清扫淋巴结，同时为放疗靶区设定提供理论依据。一般认为肺癌淋巴结转移受原发肿瘤的位置、大小、病理类型和分化程度等多种因素影响^[4]^。

### 2.1 原发肿瘤位置

对于上叶肺癌，Okada^[5]^调查了406例上叶NSCLC患者的淋巴结引流模式，所有患者均无隆突下淋巴结转移。类似地，Aokage及其同事^[6]^回顾了1, 099例上叶NSCLC患者，发现上叶NSCLC很少转移到隆突下淋巴结（1.8%, 20/1, 099）；另外对下叶肿瘤，下叶具有独特的淋巴结引流模式。Okada^[5]^已经报道47例病理N_2_患者中只有2例在没有隆突下转移的情况下出现跳跃性上纵隔转移。基于上述发现，可以认为右下叶NSCLC患者术中肺门淋巴结和隆突下均无转移时可以不清扫上纵隔淋巴结。另外法国的Riquet教授等^[7]^开展了一项研究，研究结果表明肿瘤位置无法预测淋巴结转移形式。手术治疗的NSCLC，完整的系统性纵隔淋巴结清扫仍然是唯一可接受的治疗方案。问题在于如何达成共识、如何形成指南，从而规范我们的临床治疗，使患者获益最大化。

### 2.2 原发肿瘤大小

来自大样本的研究表明原发灶肿瘤的大小是影响淋巴结转移和或远处转移及预后的极其重要因素，同样也是影响分期的一个实质性因素。

目前国内外的多项相关研究均证实肺癌淋巴结转移与肿瘤直径存在显著相关性，其中Baba等^[8]^报道的一项关于肿瘤直径与淋巴结转移相关分析显示，对肿瘤直径≤1 cm的NSCLC淋巴结转移率为3.4%。Shi等^[9]^报道研究表明对于肿瘤直径≤2 cm的NSCLC患者的淋巴结转移率为14.1%。上海市胸科医院的研究^[10]^显示肿瘤大小≤1 cm、 > 1 cm至≤2 cm和 > 2 cm至≤3 cm的淋巴结转移率分别为3.2%、14.5%和31.1%。同样结合国内外相关研究结果提示在肿瘤直径≤3 cm的肺腺癌中即使肿瘤≤1 cm，仍有可能发生淋巴结转移的可能，因此并不能单纯的根据肿瘤大小来决定是否行系统性淋巴结清扫术，同时随着肿瘤直径的增大其淋巴结转移的风险会随之增大。

### 2.3 主要组织学成分

鳞状细胞癌患者很少存在隆突下淋巴结转移，但腺癌患者显示出显著的差异。最重要的是，与鳞状细胞癌患者相比，腺癌患者更易发生N_1_和N_2_的转移。关于腺癌的主要组织学成分，贴壁生长为主型一般淋巴结状态为阴性，而微乳头和实性成分为主型淋巴结状态常常表现为阳性^[11]^。因此，需要全面揭示主要组织学成分与淋巴结转移之间的具体关系。一般对于原位腺癌和微浸润腺癌来说，不论肿瘤大小，淋巴结转移可能性几乎为零，贴壁生长为主型一般较少发生淋巴结转移，同时即使发生淋巴结转移也多为pN_1_，而对于病理亚型为实体状为主的浸润性腺癌和微乳头状为主的浸润性腺癌，其淋巴结转移率会显着高于其他病理亚型且淋巴结分期也较晚^[10]^。

目前尚无明确证据证实不同的肺腺癌亚型是否会通过影响淋巴结转移，从而进一步影响患者预后，这尚需要更多的基础研究来证实。但不同肺腺癌病理亚型对应的预后不同，意味着不同病理亚型间潜在着生物学侵袭性方面的差异。

### 2.4 肿瘤标记物

已知临床Ia期NSCLC患者血清癌胚抗原（carcino-embryonic antigen, CEA）水平与纵隔淋巴结转移有关，Hattori的研究^[12]^显示CEA水平是淋巴结转移的重要预测因子。当出现下叶临床Ⅰ期腺癌时，提示高CEA水平与上纵隔淋巴结转移有关，此外，Haruki的研究^[12]^表明CEA升高与下叶临床Ⅰ期肺腺癌中的上纵隔淋巴结转移高度相关。基于这些研究，保留正常水平CEA的下叶临床Ⅰ期NSCLC患者的上纵隔淋巴结转移似乎是合理的。然而，我们需要更多的证据来证实CEA的意义，并阐明其他肿瘤标志物的作用。

### 2.5 表皮生长因子受体（epidermal growth factor receptor, EGFR）、鼠类肉瘤病毒癌基因（kirsten rat sarcoma viral oncogene, *KRAS*）

在NSCLC患者中，EGFR通过诱导肺癌细胞来改变增殖速度，进而促进新生血管的出现，为肺癌肿瘤细胞提供浸润及转移的基础^[13]^。在亚裔肺腺癌患者中，*EGFR*突变率近50%^[14]^，而*KRAS*的突变率仅为4%-24%^[15]^。且两者突变被认为是相互独立的^[16]^。其*EGFR*突变状态对肺腺癌治疗及预后均产生重要影响，*EGFR*的存在与N_2_淋巴结跳跃转移相关，同样其往往提示更好的预后^[17]^。Li等^[18]^通过对177例亚洲肺癌患者的研究证实热点突变基因（*EGFR*和*KRAS*）与N_2_跳跃模式之间显著相关。一项荟萃分析显示，缺乏*EGFR*突变会降低手术切除的NSCLC患者的生存率和无病生存率^[19]^。有两项研究^[20, 21]^表明，手术切除的*KRAS*突变患者的生存率降低，而*EGFR*突变预后较好。但王珊等^[22]^认为*EGFR*及*KRAS*与淋巴结转移均无明显相关性。

### 2.6 血管内皮生长因子（vascular endothelial growth factor, VEGF）-C和VEGF-D及其相应的受体血管内皮生长因子受体3（vascular endothelial growth factor receptor 3, VEGFR3）

VEGF-C、VEGF-D及VEGFR3被许多人认为是肿瘤相关淋巴管发育的主要参与者，在NSCLC的肿瘤模型中，VEGF-C和EGFR3的存在导致增殖、侵袭和淋巴结转移，也有证据支持肿瘤来源的VEGF-C诱导转移前淋巴结的淋巴管发育，从而为癌细胞的到达和宿主做好准备^[23]^。

### 2.7 脏层胸膜侵犯

脏层胸膜含有极其丰富的淋巴管网，并且广泛分布于肺脏组织表面，通过穿入的肺内淋巴系统汇入支气管旁的淋巴系统，最终汇入至肺门淋巴结^[24]^，表明胸膜淋巴引流是淋巴结转移的重要途径。另外脏层胸膜侵犯的肿瘤患者的细胞可以在胸腔内发生广泛播散，一旦被膈肌或壁层胸膜重新吸收则更容易发生N_2_淋巴结转移。这也说明脏层胸膜侵犯的患者初始复发部位可能以局部复发为主。Yanagawa等^[25]^研究表明，脏层胸膜侵犯患者局部复发率远高于远处转移率，提示脏层胸膜侵犯的患者手术中是需要更彻底地进行系统淋巴结清扫，术后再根据需要选择性进行辅助放化疗或纵隔淋巴结区的照射治疗等，以防止局部复发。

## 3 NSCLC淋巴结清扫方式研究进展

淋巴结清扫对早期NSCLC的准确分期和长期生存具有重要意义。然而目前，术前很难明确判断NSCLC患者纵隔淋巴结受累情况，因此，临床上NSCLC手术过程中纵隔淋巴结清扫方式一直以来存在很大争议。根据欧洲胸外科医师协会分类，一般有5种纵隔淋巴结的处理方式：（1）系统性淋巴结清扫（systematic lymph nodes dissection, SLND）；（2）淋巴结采样（systematic lymph nodes sampling, SLNS）；（3）肺叶特异性淋巴结清扫（lobe-specific lymph nodes dissection, L-SLND）；（4）选择性淋巴结活检；（5）扩大性淋巴结清扫^[26]^；国内对于早期肺癌患者往往采用SLND、SLNS或L-SLND。究竟如何选择一直以来存有争议^[27]^。

### 3.1 SLND

SLND要求切除包括隆突下淋巴结在内的至少3站纵隔淋巴结，同时一并切除肺内和肺门淋巴结^[28]^。当右叶进行淋巴结清扫时，应切除纵隔淋巴结站点：#2R、#4R、#7、#8和#9，而对于左叶，切除纵隔淋巴结站点：#4L、#5、#6、#7、#8和#9。两侧，应将N_1_淋巴结切除作为肺切除的一部分^[29]^。

### 3.2 L-SLND

肺叶特异性淋巴结清扫主要依据病灶的不同位置从而有选择性地进行特定区域淋巴结的清扫，其理论依据主要是肺癌肿瘤细胞往往通过淋巴管路向特定的淋巴引流区域进行转移。

迄今为止有几项回顾性研究旨在确定肺叶特异性淋巴结清扫术是否可以作为标准治疗方案的选择，并且可以提供临床获益。然而，不同的研究选择不同的肺叶特异性淋巴结清扫术方案。Hishida等^[30]^的方案为：对于右上肺肿瘤，切除上纵隔淋巴结（#2R和#4R），同时左上叶切除上纵隔淋巴结和主动脉旁淋巴结（#4L、#5和#6）。对于上叶保留下纵隔淋巴结（#7、#8和#9）。对于下叶，切除下纵隔淋巴结（#7、#8和#9），而保留上纵隔淋巴结和或主动脉旁淋巴结。Adachi等^[31]^采用了相同的方案。Ishiguro等^[32]^的研究中采用了术中冰冻切片，右上肺肿瘤切除上纵隔淋巴结，左上肺切除主动脉旁和上纵隔淋巴结，下叶切除下纵隔淋巴结。手术切除淋巴结时应进行冰冻切片分析，如果阳性，则进行SLND。

### 3.3 SLNS

通过术前或术中发现为引导，移除一个或多个淋巴结。Darling^[29]^建议对于右肺肿瘤，行第2R、4R、7、10R组采样，左侧肿瘤建议行第5、6、7、10L进行采样。目前NCCN指南仍推荐至少采集或切除3站淋巴结。Takizawa等^[33]^报道临床Ⅰ期NSCLC行系统淋巴结清扫组术后的5年总体生存率为78.0%，淋巴结采样组术后的5年总体生存率约为76.2%，临床Ⅰ期NSCLC患者SLNS与SLND预后相当。Stiles等^[34]^报道Ia期NSCLC患者楔形切除术中行SLNS相比未采样的患者出现局部复发率更低，楔形切除手术联合SLNS治疗并不会增加手术并发症及住院时间，但却可降低局部复发率，同时提高生存率。

### 3.4 淋巴结清扫数目的选择

多数研究证实淋巴结清扫数量的可变性应根据研究的设计类型、肿瘤分期和淋巴结站数来定。这种变化可能与预后价值有关，但也可能与解剖变异相关。为了分析在SLND中完成淋巴结清扫数量的变异及其对一系列NSCLC手术预后的影响，Riquet教授等^[7]^完成了该项研究，研究表明肺癌根治术中SLND的淋巴结的清扫数量呈正态分布，对术后总体生存率无显著影响。从而表明术中清扫淋巴结的数量的最合适数目是任意的。但Rique教授的建议是依据解剖结构行彻底的SLND。另有研究认为术后患者生存率与清扫的淋巴结数量相关，淋巴结清扫的数量决定了更准确的淋巴结分期相关及指导后续治疗^[35, 36]^。Ludwig等^[37]^通过对SEER数据库的分析研究，认为肺叶切除患者手术中切除淋巴结个数在13个-16个之间，患者的生存获益最高。Samayoa等^[35]^建议，早期NSCLC的肺叶切除治疗应包括至少10个淋巴结的清扫。周教授^[38]^认为在早期NSCLC中淋巴结采样术大于3站可显著提高总生存率。

### 3.5 纵隔淋巴结清扫的不同方式的临床价值

目前随着越来越多的早期肺癌的发现，人们正不断探索这部分肺癌病人的最佳切除方式，即如何最大可能减少手术创伤和生存获益最大。目前在早期肺癌的淋巴结切除范围、个数尚无统一标准。

SLND与SLNS相比，并不能改善早期NSCLC患者的生存率。不过，SLND也并未增加死亡率或发病率，SLND仍被推荐用于早期NSCLC，因为它可以通过增加淋巴结采集来提高对隐匿性N_2_疾病的识别来增强分期准确性^[39, 40]^，美国外科肿瘤学会（American College of Surgical Oncologists Group, ACOSOG）Z0030试验^[41]^表明SLND与SLNS相比平均手术时长仅延长14 min，术后平均引流量仅增加200 mL，术后住院时间仅延长1天。Izbicki^[42]^和Graham^[43]^认为，SLND应该常规用于早期NSCLC。一方面，SLND是否会增加术后并发症发生率并不明确，以及诸如对总生存率和无病生存率结果的影响仍然存在争议。另一方面，L-SLND和SLNS是否可以缩短整个治疗时间并具有有效性、可靠性和适用性仍然不是很清楚。此外，有研究报道L-SLND增加了患者的复发可能。尤其是NSCLC的淋巴结转移特点存在多发性和跳跃性，早期NSCLC可出现淋巴结微转移从而导致术后局部复发的风险增高^[44]^。

来自日本的Adachi等^[45]^开展了一项研究，该研究表明，L-SLND在病理分期准确性和术后生存上均不劣于SLND，提示L-SLND可作为早期NSCLC的手术治疗的一个标准方式。Shapiro等^[46]^也得出相似结论，其认为早期NSCLC行SLND与L-SLND的患者其术后5年无瘤生存率和局部复发率之间无差异，而且L-SLND的手术并发症相对更少。

## 4 结语与展望

虽然淋巴结清扫方式存在诸多争议，目前术中指导N_2_淋巴结清扫程度的前哨淋巴结成像研究尚未完善。特定肺叶-淋巴引流图谱已变得越来越可靠和实用，但仍有几个重要问题需要回答。如肿瘤位置、大小、突变情况、分化程度和肿瘤标志物对淋巴结转移的复杂影响尚不清楚。目前美国（CALBG140503）和日本（JCOG0802/WJOG4607L）正在开展比较≤2 cm NSCLC的肺叶切除和局限性切除的随机对照试验。因此，在这些随机对照研究结果出来前，早期NSCLC的治疗选择仍存在疑问。那么，外科医生在选择手术方式时必须考虑最新的研究证据和患者的临床情况，依据患者的身体条件情况、病灶影像特征及术前术中冰冻切片信息对患者施行个体化的淋巴结清扫策略。
